# Dietary and lifestyle associations with microbiome diversity

**DOI:** 10.1186/s13099-022-00525-w

**Published:** 2022-12-23

**Authors:** Katherine M. Watson, Kyla N. Siemens, Sudarshan Anand, Ivy H. Gardner, Thomas J. Sharpton, Elizabeth N. Dewey, Robert Martindale, Christopher A. Gaulke, Vassiliki Liana Tsikitis

**Affiliations:** 1 Department of Surgery, Oregon Health & Science University, 3181 SW Sam Jackson Park Rd, Mail Code: L223A Portland, OR US; 2 Department of Cell, Developmental & Cancer Biology, Oregon Health & Science University, Portland, OR US; 3 Department of Microbiology, Oregon State University, Corvallis, OR US; 4 Department of Statistics, Oregon State University, Corvallis, OR US; 5 Department of Pathobiology, University of Illinois Urbana-Champaign, Urbana, IL US; 6 Carl R. Woese Institute for Genomic Biology, University of Illinois Urbana-Champaign, Urbana, IL US

## Abstract

**Background:**

Microbial dysbiosis has been closely linked with colorectal cancer development. However, data is limited regarding the relationship of the mucosal microbiome, adenomatous polyps and dietary habits. Understanding these associations may elucidate pathways for risk stratification according to diet.

**Results:**

Patients undergoing screening colonoscopy were included in our prospective, single center study and divided into adenoma or no adenoma cohorts. Oral, fecal, and mucosal samples were obtained. Microbial DNA was extracted, and amplicon libraries generated using primers for the 16S rRNA gene V4 region. Patient and dietary information was collected. Of 104 participants, 44% presented with polyps, which were predominantly tubular adenomas (87%). Adenoma formation and multiple patient dietary and lifestyle characteristics were associated with mucosal microbiome diversity. Lifestyle factors included age, body mass index, adenoma number, and dietary consumption of red meats, processed meats, vegetables, fruit, grain, fermented foods and alcohol.

**Conclusion:**

In this study we showed associations between dietary habits, adenoma formation and the mucosal microbiome. These early findings suggest that ongoing research into diet modification may help reduce adenoma formation and subsequently the development of CRC.

**Supplementary Information:**

The online version contains supplementary material available at 10.1186/s13099-022-00525-w.

## Introduction

Colorectal cancer (CRC) is a leading cause of cancer-related mortality and has been associated with environmental, nutritional and behavioral factors as well as with age [1–3]. Mounting data suggests that the enteric microbiome may be associated with CRC development. While the majority of an individual’s microbiome is relatively stable outside of extremes of age [4], human factors, such as diet, alcohol consumption and exercise have the potential to reshape the gut microbiome [5, 6], and therefore may serve as modifiable factors to help prevent the development of CRC. Since CRC arises out of adenomatous polyps [7], identifying changes in the microbiome associated with adenomas may help understand some of the early changes associated with tumorigenesis. We have previously shown that the colonic mucosal microbiome in patients with adenomas is distinct from patients without adenomatous polyps [8]. In this study, we examine the interplay between patient clinical factors, their oral, fecal and mucosal microbiome diversity and adenoma burden. Using the same cohort of patients undergoing screening colonoscopy [8], we evaluated the effects of health and dietary characteristics on the diversity of the microbiome, and describe associations among adenoma and non-adenoma formers and their health and dietary practices.

## Methods

### Subject enrollment and data analysis

**Fig. 1:**
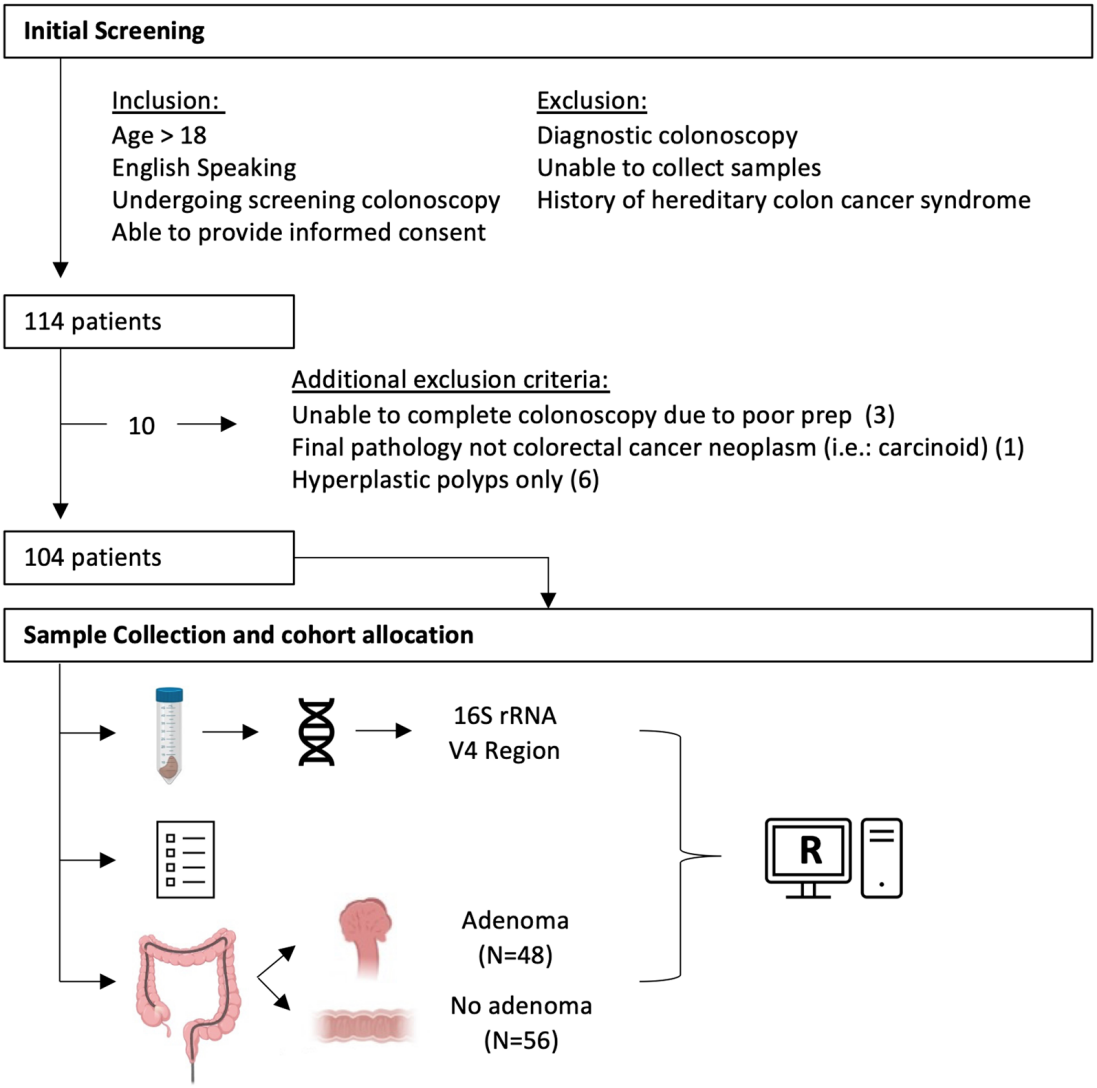
Study Flow Diagram. Patients undergoing screening colonoscopy were screed for inclusion in the study. Of the 114 patients enrolled, 10 were excluded. Samples and data from the remaining 104 patients were used for data analysis. Stool samples were collected prior to bowel preparation using the Zymo Research DNA/RNA Shield Collection Tube and Tube. Samples were processed using the DNeasy PowerSoil Pro isolation kit. Sequencing was performed on an Illumina MiSeq instrument by amplification of the V4 region of the 16S rRNA. Questionnaires were obtained including patient medical history, dietary information and habits. Data was analyzed using R statistical software. To determine how other host metadata parameters influenced microbiome diversity, we performed a constrained ordination analysis. A stepwise model construction procedure for constrained ordination was then used to select a reduced model with P-value permutation

Subjects scheduled to undergo screening colonoscopy at Oregon Health and Science University were prospectively enrolled in the study from October 2018–2019 following informed consent (Institutional Review Board #17350). Oral samples and fecal samples prior to bowel prep were obtained. The patient inclusion flowchart is included in Fig. 1. Patient information was obtained from the electronic medical record and patient questionnaires (Additional file 1and 2). Regular activity was defined as equal to or greater than 150-min of moderate-intensity or 75-min of vigorous-intensity aerobic physical activity each week per US Department of Health & Human Services standard [9].

### Sequencing and microbiome analysis

Stool, tissue, and oral swab samples were obtained from patients and stored and processed as previously described [10.1097/SLA.0000000000005261]. Briefly, DNA was extracted from fecal, oral swab, and mucosal samples using the DNeasy PowerSoil Pro kit according to the manufacturer’s protocol with the addition of a 10-min incubation at 65 °C immediately before bead beating to facilitate bacterial lysis. This incubation was followed by bead beating on the highest setting for 10-min using Vortex Genie 2 and a 24-sample vortex adaptor. Isolated DNA (1µL) was used to amplify the 16S rRNA V4 region in triplicate as previously described using the 515f and 806r primers [10.1073/pnas.1000080107 ; 10.1038/ismej.2012.8]. Amplicon libraries were quantified and 200 ng of each library was pooled, purified using the QIAquick PCR Purification Kit, and sequenced on an Illumina MiSeq instrument (300 bp. Sequence reads were input into DADA2 [29] for quality filtering, amplicon sequence variant (ASV) calling (default parameters), and taxonomic assignment against the Silva database (v128). Non-prokaryotic reads were filtered from our dataset and raw ASV tables were rarefied to a depth of 10,000 counts using R and vegan [10.1111/j.1654-1103.2003.tb02228.x]. Samples with less than 10,000 sequences were removed prior to rarefaction.

### Statistical analyses

We applied constrained metric scaling (vegan::capscale; distance = “euclidean”) followed by bidirectional stepwise model selection (vegan::ordistep)and environmental fitting (vegan::envfit) to log transformed (vegan::decostand) rarefied fecal, mucosal, and oral genera abundance data (R v4.1.1), individually, (https://github.com/chrisgaulke/ohsu_combined_adenoma_data) to identify patient parameters that best explain microbiome diversity (Table 1). False discovery rate was controlled at 0.1 for both envfit and ordistep analyses using with R::p.adjust(method = “fdr”).The overall significance of each model and the significance of model terms, was assessed using an ANOVA-like permutational test (vegan::anova.cca). The variation explained by each model was calculated using vegan:: RsquareAdj. Continuous variables were compared using a t-test for parametric variables and a Mann–Whitney U test for nonparametric variables. Categorical variables were compared using Chi-square test or Fisher exact test given distribution.

**Table 1:** Redundancy analysis coupled with model selection identifies lifestyle factors that associate with microbiome diversity

	F	p-value
Fecal		
Bristol Score	1.27	1.80E-02
Age	1.31	2.70E-02
Processed Meat	1.55	3.30E-02
Fruit	1.85	1.10E-02
Grain	1.55	3.80E-02
Regular Activity	2.52	1.00E-03
Alcohol use	1.57	3.20E-02
Diabetes	1.36	2.70E-02
Vitamin D	1.75	1.10E-02
Hormone Therapy	1.79	1.40E-02
Oral		
Bristol Score	1.45	8.00E-03
BMI	3.31	2.00E-03
Red Meat	1.66	6.80E-02
Calcium	2.41	1.50E-02
Mucosal		
Age	1.41	1.30E-02
Adenoma	2.44	5.00E-03
Fruit	1.89	7.00E-03
Diabetes	1.98	2.00E-03
Vitamin D	1.45	4.60E-02
Hormone Therapy	1.75	1.70E-02

F-statistic and *p-value* generated using an ANOVA-like permutation test (R::vegan::anova.cca) for each predictor retained after model selection. False discover rate was controlled at 0.1. Factors evaluated were age; bristol score; gender; body mass index (BMI); regular activity; number of polyps; number of adenomas; consumption of red meat, vegetable, fruit, grain, processed meat, fermented food, alcoholic beverages; diseases including: gastroesophageal reflux disease (GERD), cancer, autoimmune disease, diabetes, other gastrointestinal disorders; and medications including: ASA, NSAID, Vitamin D, Vitamin E, Calcium, metformin, hormone replacement therapy and probiotics

### Data availability

The raw data files from this work are published at the SRA project numbers PRJNA650009 and PRJNA745994. The metadata, ASV, and taxonomy tables are available at https://github.com/chrisgaulke/adenoma_cca.

## Results

One-hundred-four patients underwent screening colonoscopy with a mean age of 60 years (range 41–78 years, SD ± 8.7). Adenomatous polyps were identified in 46% of participants and were most commonly located in the ascending colon (58%). The vast majority of adenomas were tubular adenomas (87% vs 2% tubulovillous and 11% sessile). Cohorts with and without adenomas were similar in dietary practices and patient characteristics with the exception of smoking (25% controls, 48% adenoma formers, *p* = 0.015) and regular activity (79% controls, 58% adenoma formers, *p* = 0.026) [8]. To identify associations between patient characteristics and microbiome diversity we coupled redundancy analysis with stepwise model selection. Overall, oral (F_(12,71)_ = 1.71; p = 0.001; R^2^ = 0.09), fecal (F_(21,67)_ = 1.47; p = 0.001; R^2^ = 0.1), and mucosal (F_(12,77)_ = 1.45; p = 0.001; R^2^ = 0.06) microbial communities were moderately associated with host dietary habits and health. Oral microbial community diversity varied significantly with Bristol score, patient BMI, calcium supplementation, red meat consumption, and consumption of fermented foods (Table 1). There was little overlap between the factors that significantly associated with oral microbiome diversity and those that associated with fecal and mucosal diversity. However, fecal, and mucosal microbial communities were both substantially impacted by age, fruit consumption, diabetes, calcium supplementation and hormone replacement therapy. Fecal diversity also associated with alcoholic beverage and grain consumption, but while observed in mucosal compartments as well, these associations did not reach significance (Table 1). Consistent with our previous work [8], only mucosal microbiome diversity linked with adenoma burden (Table 1).

**Fig. 2:**
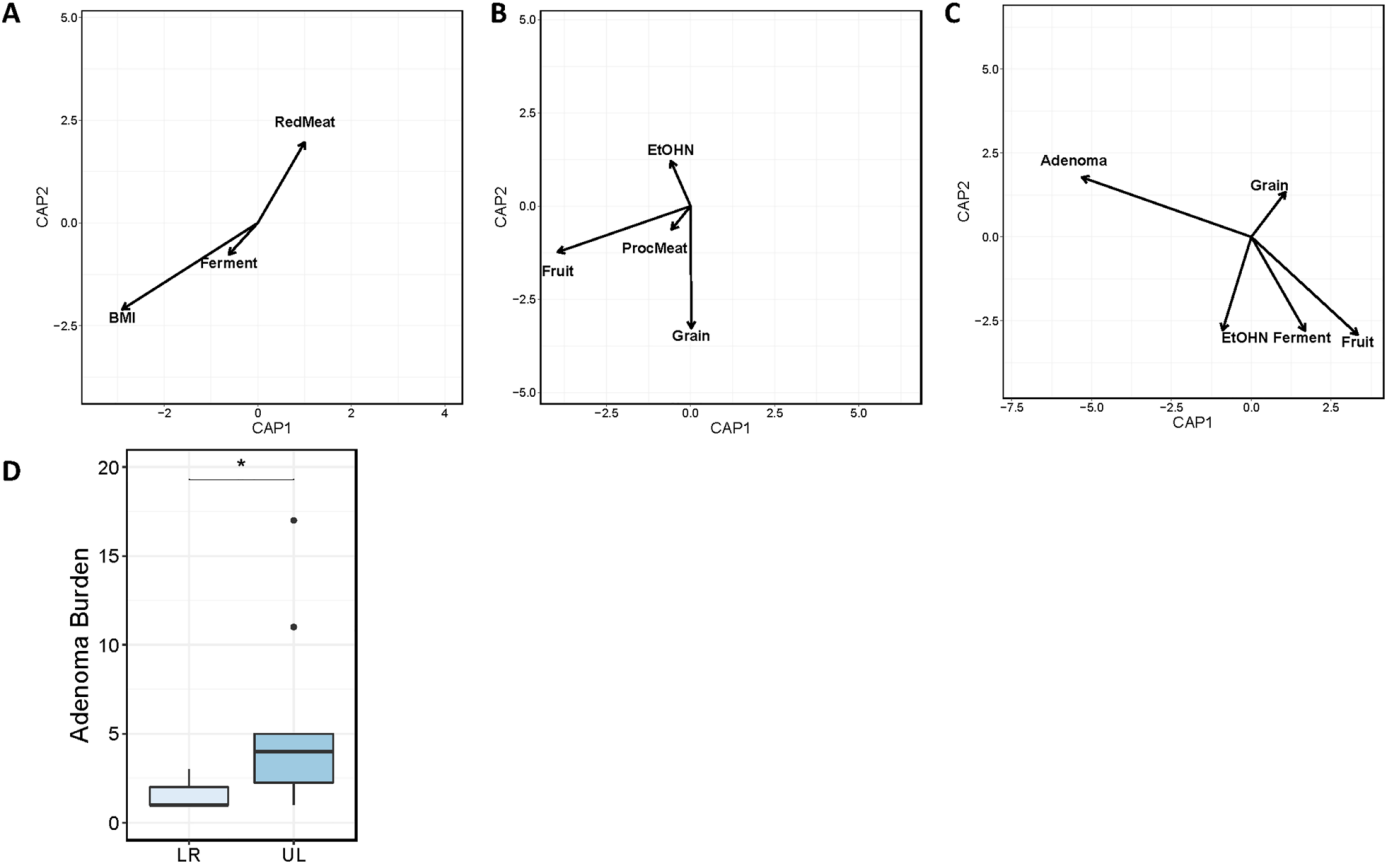
Patient lifestyle is associated with microbiome diversity. Constrained ordinations of **A** oral, **B** fecal, **C** mucosal diversity overlaid with vectors (arrows) representing the direction and magnitude of the correlation of between parameter and the primary axes of microbiome diversity variation. Factors shown include body mass index (BMI); fermented food (Ferment), red meat (RedMeat), fruit, grain, and alcoholic beverage (EtOHN) consumption; and adenoma number (Adenoma). **D** A boxplot of adenoma number per patient in patients with adenomas in the lower right (LR) and upper left (UL) quadrants of the mucosal microbial diversity ordination. An * denotes p < 0.05

To evaluate associations between patient characteristics and health behaviors and microbiome diversity we fit each patient and health factor that our model selection procedure identified as contributing to microbiome diversity to a constrained ordination of microbiome diversity (Table 2). This allowed us to identify linear association between microbiome diversity and patient characteristics and how these associations were related. Oral microbiome diversity correlated with BMI (R^2^ = 0.27, *p* = 1.0 × 10^− 3^), red meat consumption (R^2^ = 0.1019, *p* = 0.02) and calcium supplementation (R^2^ = 0.08, *p* = 2.0 × 10^− 3^; Fig. 2A). Instead, fruit and grain consumption (R^2^ = 0.22, *p* = 1.0 × 10^− 3^) dominated the correlation with fecal microbial diversity (Fig. 2B). In addition, regular activity (R^2^ = 0.15, *p* = 1.0 × 10^− 3^), diabetes (R^2^ = 0.21, *p* = 1.0 × 10^− 3^), Bristol stool scale (R^2^ = 0.29, p = 1.0 × 10^− 3^), age (R^2^ = 0.01, *p* = 1.0 × 10^− 3^), vitamin D consumption (R^2^ = 0.04, *p* = 3.1 × 10^− 2^) and hormonal therapy (R^2^ = 0.04, *p* = 1.7 × 10^− 2^) were also associated with fecal microbiome diversity.

**Table 2:** Environmental fitting identifies linear associations between microbial diversity and host lifestyle

	R^2^	p-value
Fecal		
Fruit	0.22	1.00E-03
Grain	0.14	3.00E-03
Bristol	0.29	1.00E-03
Age	0.11	1.00E-03
Regular Activity	0.15	1.00E-03
Diabetes	0.21	1.00E-03
Vitamin D	0.04	3.10E-02
HRT	0.04	1.70E-02
Oral		
Bristol Score	0.51	1.00E-03
BMI	0.27	1.00E-03
Red Meat	0.10	2.00E-02
Calcium	0.08	2.00E-03
Mucosal Tissue		
Adenoma Number	0.32	1.00E-03
Fruit	0.20	1.00E-03
Fermented Foods	0.11	4.00E-03
Alcohol Use	0.09	1.70E-02
Age	0.26	1.00E-03
Diabetes	0.29	1.00E-03
HRT	0.05	1.20E-02

An R^2^ and *p-value* generated from an environmental fit of each predictor to the primary axes of variation (CAP 1 and 2). False discover rate controlled at 0.1

*HRT* Hormone replacement therapy

There were some similarities between the mucosal microbiome diversity from that of the fecal microbiome. Fruit consumption was again associated with microbiome diversity (R^2^ = 0.20, *p* = 1.0 × 10^− 3^) and there was a trend in grain consumption though not significant (R^2^ = 0.30, *p* = 0.26; Fig. 2C). Fermented foods and alcohol consumption were also significantly associated with mucosal microbiome diversity (R^2^ = 0.11, *p* = 4.0 × 10^− 3^, R^2^ = 0.09, *p* = 0.17). Other patient factors such as age (R^2^ = 0.26, *p* = 1.0 × 10^− 3^), diabetes (R^2^ = 0.29, *p* = 1.0 × 10^− 3^), and hormonal therapy (R^2^ = 0.51, *p* = 0.12) were also associated. The greatest association between diversity and the mucosal microbiome was adenoma presence (R^2^ = 0.32, *p* = 1.0 × 10^− 3^). In fecal communities, the vectors of association between alcoholic beverage and grain consumption and microbiome diversity suggest that these lifestyle factors may have opposing effects on the microbiome. Similarly, in mucosal samples, total adenoma burden and consumption of fermented foods and fruit exhibit distinct opposing effect on microbiome diversity, suggesting that individuals with high fruit and fermented food intake tend to manifest patterns of microbiome diversity that associate with lower adenoma burden. Supporting this observation, patients in the ordination quadrant associated with higher fruit consumption (lower right) had significantly fewer adenomas when compared in the quadrant opposite (upper left; Fig. 2D).

## Discussion

Diet and exercise have been shown to affect CRC risk [1–3]. We recently demonstrated that specific microbial community compositions associate with adenoma formation. However, the factors that underly the assembly of a microbiota that promotes CRC or adenoma formation remains unresolved. Here we have identified host lifestyle factors including consumption of specific food types that promote mucosal microbiome diversity associated with lower levels of adenoma burden [8]. Like our previous work, the only association between microbiome diversity and adenomas was that of the mucosal microbiome and while we appreciated differences between human factors and microbiome diversity based on the sampling site (oral, fecal, mucosal). We also found that there were some similarities, in particular, fruit, grain and fermented food intake, appeared to be associated with mucosal microbiome diversity. Diabetes, hormone replacement therapy, vitamin D supplementation, alcohol consumption, age and regular activity were also correlated with diversity of the mucosal microbiome.

Overall, these findings are consistent with epidemiological associations with colorectal cancer [3]. In our study were not able to disentangle the complex relationships between the different variables included, however, we can appreciate a relationship between diet, the mucosal microbiome and adenomas. However, while dietary habits and human factors have been shown to affect colorectal adenoma and cancer risk, mechanisms to explain this phenomenon are not yet elucidated, though the microbiome presents as a reasonable conduit for the deleterious effects of human habits. The interplay between patient habits, the microbiome and adenomas, is complex, however, even with our cohort we see relationships suggesting that these interactions are real, and may present an impactful point for intervention. To fully understand these relationships would take a multi-institutional effort, one which further builds on these data to show that the mucosal microbiome may be altered, that interventions are affordable and that compliance with such interventions may be sustainable for patients with high compliance.

## Conclusions

In this study we showed associations between dietary habits, adenoma formation and in particular the mucosal microbiome. It is suggested that diet modification shapes mucosal microbiome and may pose a modifiable risk factor for adenoma formation and the development of colorectal neoplasia.

## Supplementary Information

**Additional file 1**. Patient data collection form.

**Additional file 2**. Dietary data collection form.

## Data Availability

The datasets generated and analyzed during the current study are available at https://github.com/chrisgaulke/ohsu_combined_adenoma_data.

## References

[1] Park SY, Boushey CJ, Wilkens LR, Haiman CA, Le Marchand L (2017). High-quality diets associate with reduced risk of colorectal cancer: analyses of diet quality indexes in the multiethnic cohort. Gastroenterology.

[2] Nigro ND, Bull AW (1987). Prospects for the prevention of colorectal cancer. Dis Colon Rectum.

[3] Dekker E, Tanis PJ, Vleugels JLA, Kasi PM, Wallace MB (2019). Colorectal cancer. Lancet.

[4] Faith JJ, Guruge JL, Charbonneau M, Subramanian S, Seedorf H, Goodman AL (2013). The long-term stability of the human gut microbiota. Science.

[5] Nelson H, Chia N (2019). Gut Microbiome and Colon Cancer: A Plausible Explanation for Dietary Contributions to Cancer. J Am Coll Surg.

[6] David LA, Maurice CF, Carmody RN, Gootenberg DB, Button JE, Wolfe BE (2014). Diet rapidly and reproducibly alters the human gut microbiome. Nature.

[7] Leslie A, Carey FA, Pratt NR, Steele RJC (2002). The colorectal adenoma ± carcinoma sequence. British J Surg.

[8] Watson KM, Gardner IH, Anand S, Siemens KN, Sharpton TJ, Kasschau KDP (2021). Colonic microbial abundances predict adenoma formers. Ann Surg.

[9] Piercy KL, Troiano RP, Ballard RM (2018). The Physical Activity Guidelines for Americans. JAMA..

