# Supplementary material for: Dietary and lifestyle associations with microbiome diversity
Source: Gut Pathog. 2022 Dec 23;14:49. doi: 10.1186/s13099-022-00525-w (PMC9784278; doi:10.1186/s13099-022-00525-w)
Supplement: Supplementary file 1 — Additional file 1. Patient data collection form. [file 13099_2022_525_MOESM1_ESM.docx]

**Patient Data Collection Form**

Date of Completion: __________________

Study ID: __________________

1. Date of birth (DD/MM/YYYY)? ________________________

1. Gender Male Female
2. What is your race? White

Black / African American

Native American / Alaska Native

Asian

Native Hawaiian / Pacific Islander

Other (specify): ____________________

1. What is your ethnicity? Hispanic or Latino

Non-Hispanic or Latino

1. Height (cm) ________________________
2. Weight (kg) ________________________
3. BMI Calculated by study staff____
4. Are you a current/active smoker? __YES__________NO____

If YES, number of cigarettes/day? ________________________

1. Are you a prior smoker? __YES__________NO____
2. Do you use alcohol? __YES__________NO____

If YES, number of drinks/week? ________________________

1. Do you or your family have a history of colorectal cancer?

__YES (specify): __________________________________________________________

__NO

1. Do you or your family have a history other cancers?

__YES (specify): __________________________________________________________

__NO

1. Do you or your family have a history of inflammatory bowel disease?

__YES (specify): __________________________________________________________

__NO

1. Do you have a history of colonic polyps?

__YES (specify): __________________________________________________________

__NO

1. Other pertinent medical conditions?

__YES (specify): __________________________________________________________

__NO

1. Do you have a history of previous colonic surgeries?

__YES (specify): __________________________________________________________

__NO

1. Current medications or supplements?
   - Aspirin
   - Other NSAIDS (ibuprofen, Naproxen)
   - Vitamin D
   - Vitamin E
   - Calcium
   - Metformin
   - Hormone replacement therapy
   - Probiotics
   - Other (specify): ­­­­­­­­­­­­­­­­­­­­­­­­­­­­___________________________________________________
2. Current Antibiotic Usage (within the last 90 days)

__YES (specify): __________________________________________________________

__NO

1. Describe your red meat consumption (beef, pork, lamb, or veal). (1 portion = 4 ounces or approximately the size of a deck of cards. Examples: 4 oz steak, ¼ lb hamburger)

- None
- More than 1 portion daily
- 1 portion daily
- 1 to 2 portions per week
- 1 to 2 portions per month

1. Describe your processed meat consumption. (processed meat = bacon, ham, sausage, salami, pepperoni, hot dogs, spam, bologna)

- None
- Daily
- 2 to 3 times per week
- Once weekly
- Once monthly

1. Describe your vegetable consumption. (1 serving of vegetables = 1 cup of raw vegetables or ½ cup of cooked vegetables)

- None
- More than 3 servings daily
- 1 to 3 servings daily
- 1 to 3 servings weekly
- 1 to 3 servings monthly

1. Describe your fruit consumption. (1 serving fruit = 1 cup raw or canned fruit, small apple, large banana, medium grapefruit, large orange, 1 cup 100% fruit juice)

- None
- More than 3 servings daily
- 1 to 3 servings daily
- 1 to 3 servings weekly
- 1 to 3 servings monthly

1. Describe your whole grain consumption (whole wheat bread, oats, brown rice, quinoa, wheat, barley, farro, millet, buckwheat, couscous, etc.). (1 serving of grains = ½ cup cooked oatmeal, 1 slice bread, ½ cup cooked brown rice, ½ cup cooked whole grain pasta)

- None
- More than 3 servings daily
- 1 to 3 servings daily
- 1 to 3 servings weekly
- 1 to 3 servings monthly

1. How often do you eat fermented foods? (Examples of fermented foods include yogurt, kefir, sauerkraut, kimchi, kombucha, tempeh, miso, and buttermilk)

- Never
- Daily
- 2 to 3 times per week
- Once weekly
- Once monthly

1. Aerobic Activity (cardio activity)(minutes/week) ____________________________
2. Type of colonic prep for colonoscopy procedure?
   - Golytely
   - Suprep
   - MoviPrep
   - Other (specify): ______________________________________

**CRF Form Completion:**

Form completed by: ______________________________________

Date/time of completion: ______________________________________

**CRF Form Review:**

P.I. signature: ______________________________________

Date of review: ______________________________________
