# Supplementary material for: Dietary and lifestyle associations with microbiome diversity
Source: Gut Pathog. 2022 Dec 23;14:49. doi: 10.1186/s13099-022-00525-w (PMC9784278; doi:10.1186/s13099-022-00525-w)
Supplement: Supplementary file 2 — Additional file 2. Dietary data collection form. [file 13099_2022_525_MOESM2_ESM.docx]

**Dietary Data Collection Form**

Date of Completion: __________________

Study ID: __________________

1. Describe your red meat consumption (beef, pork, lamb, or veal). (1 portion = 4 ounces or approximately the size of a deck of cards. Examples: 4 oz steak, ¼ lb hamburger)

- None
- More than 1 portion daily
- 1 portion daily
- 1 to 2 portions per week
- 1 to 2 portions per month

1. Describe your processed meat consumption. (processed meat = bacon, ham, sausage, salami, pepperoni, hot dogs, spam, bologna)

- None
- Daily
- 2 to 3 times per week
- Once weekly
- Once monthly

1. Describe your vegetable consumption. (1 serving of vegetables = 1 cup of raw vegetables or ½ cup of cooked vegetables)

- None
- More than 3 servings daily
- 1 to 3 servings daily
- 1 to 3 servings weekly
- 1 to 3 servings monthly

1. Describe your fruit consumption. (1 serving fruit = 1 cup raw or canned fruit, small apple, large banana, medium grapefruit, large orange, 1 cup 100% fruit juice)

- None
- More than 3 servings daily
- 1 to 3 servings daily
- 1 to 3 servings weekly
- 1 to 3 servings monthly

1. Describe your whole grain consumption (whole wheat bread, oats, brown rice, quinoa, wheat, barley, farro, millet, buckwheat, couscous, etc.). (1 serving of grains = ½ cup cooked oatmeal, 1 slice bread, ½ cup cooked brown rice, ½ cup cooked whole grain pasta)

- None
- More than 3 servings daily
- 1 to 3 servings daily
- 1 to 3 servings weekly
- 1 to 3 servings monthly

1. How often do you eat fermented foods? (Examples of fermented foods include yogurt, kefir, sauerkraut, kimchi, kombucha, tempeh, miso, and buttermilk)

- Never
- Daily
- 2 to 3 times per week
- Once weekly
- Once monthly
